# Effect of neglecting passive spinal structures: a quantitative investigation using the forward-dynamics and inverse-dynamics musculoskeletal approach

**DOI:** 10.3389/fphys.2023.1135531

**Published:** 2023-05-31

**Authors:** Laura Meszaros-Beller, Maria Hammer, Syn Schmitt, Peter Pivonka

**Affiliations:** ^1^ School of Mechanical, Medical and Process Engineering, Queensland University of Technology, Brisbane, QLD, Australia; ^2^ Centre for Biomedical Technologies, Queensland University of Technology, Brisbane, QLD, Australia; ^3^ Institute for Modelling and Simulation of Biomechanical Systems, University of Stuttgart, Stuttgart, Germany; ^4^ Stuttgart Center for Simulation Science (SC SimTech), University of Stuttgart, Stuttgart, Germany

**Keywords:** spine biomechanics, musculoskeletal modelling, inverse-dynamics, forward-dynamics, passive soft tissues

## Abstract

**Purpose:** Inverse-dynamics (ID) analysis is an approach widely used for studying spine biomechanics and the estimation of muscle forces. Despite the increasing structural complexity of spine models, ID analysis results substantially rely on accurate kinematic data that most of the current technologies are not capable to provide. For this reason, the model complexity is drastically reduced by assuming three degrees of freedom spherical joints and generic kinematic coupling constraints. Moreover, the majority of current ID spine models neglect the contribution of passive structures. The aim of this ID analysis study was to determine the impact of modelled passive structures (i.e., ligaments and intervertebral discs) on remaining joint forces and torques that muscles must balance in the functional spinal unit.

**Methods:** For this purpose, an existing generic spine model developed for the use in the *demoa* software environment was transferred into the musculoskeletal modelling platform *OpenSim*. The thoracolumbar spine model previously used in forward-dynamics (FD) simulations provided a full kinematic description of a flexion-extension movement. By using the obtained *in silico* kinematics, ID analysis was performed. The individual contribution of passive elements to the generalised net joint forces and torques was evaluated in a step-wise approach increasing the model complexity by adding individual biological structures of the spine.

**Results:** The implementation of intervertebral discs and ligaments has significantly reduced compressive loading and anterior torque that is attributed to the acting net muscle forces by −200% and −75%, respectively. The ID model kinematics and kinetics were cross-validated against the FD simulation results.

**Conclusion:** This study clearly shows the importance of incorporating passive spinal structures on the accurate computation of remaining joint loads. Furthermore, for the first time, a generic spine model was used and cross-validated in two different musculoskeletal modelling platforms, i.e., *demoa* and *OpenSim*, respectively. In future, a comparison of neuromuscular control strategies for spinal movement can be investigated using both approaches.

## 1 Introduction

Accurate estimation of joint loading is of high significance to study the biomechanics of the spine. Several musculoskeletal (MSK) spine models have been introduced in literature (de Zee et al., 2007; Christophy et al., 2012; Bruno et al., 2015; Rupp et al., 2015; Cazzola et al., 2017; Mörl et al., 2020; Guo et al., 2021; Silvestros et al., 2022; Meszaros-Beller et al., 2023) with the goal to predict muscle forces, muscle activation patterns and internal loading conditions during human movement.

There are two multibody (MB) approaches typically used to determine these quantities: the forward-dynamics (FD) and the inverse-dynamics (ID) approach. Both approaches aim to provide the optimal solution to the redundancy problem, a mathematical overdeterminacy of the MSK system arising from a greater number of muscles crossing a joint than number of degree of freedom (DOF) specifying the joint movement (Pandy, 2001). Which approach is appropriate to be used depends on the intended purpose of the modelling study.

The main goal of the FD approach is to gain an intrinsic understanding of movement control, i.e., how the nervous system and the muscles use sensory information to produce a coordinated movement. FD works in a forward sense in which muscle forces governed by the underlying muscle-tendon dynamics act on the skeletal geometry in response to muscle stimulation initiated by the central nervous system. The predicted movement is the result of a dynamic interplay of all modelled structural components. The drawback of the FD approach is that it requires accurate modelling of all load-bearing structures, i.e., muscles, ligaments, the intervertebral disc (IVD), and the control scheme predicting individual muscle stimulation. Applied on the spine, this is particularly difficult regarding the high number of articulations and muscles and ligaments involved in stabilising the mechanically instable multi-joint structure of the upright human spine (Smit, 2020).

Due to the structural complexity of the spine, the FD approach has been rarely used to simulate spine biomechanics but has risen to new heights in recent years. A detailed lumbar spine model with a lumped upper body was introduced by Rupp et al. (2015), validated with respect to its passive stiffness properties in a lying posture (Mörl et al., 2020) and recently extended to include articulated thoracic vertebrae in order to quantify the load sharing between individual biological structures of the functional spinal unit under gravity (Meszaros-Beller et al., 2023). Another FD lumbar spine model was presented in Damm et al. (2020). In this study, ligament material properties were reverse engineered from experimental stiffness measurements conducted on cadaveric functional spinal units (Heuer et al., 2007). Both, Damm et al. (2020) and Mörl et al. (2020) highlighted the importance of having physiologically accurate material properties of passive spinal structures, i.e., of ligaments in particular. Further, Müller et al. (2021) used the FD approach to undertake a quantitative investigation of the effect of varying lumbar lordosis angle and muscle activation on the load distribution. Finally, Guo et al. (2021) recently presented a full spine model with an articulated cervical, thoracic and lumbar region to demonstrate the muscle activity-dependent change in intra-abdominal pressure and and its unloading effect on the spine.

On the other hand, the ID approach serves a more descriptive, analytic purpose: the computation of generalised net joint forces and torques for a specific motion task of interest (Pandy, 2001) and the corresponding net contribution of muscle forces to the joint loads which are assumed to compensate the remaining net joint forces and torques from the ID analysis. Thus, ID results are often interpreted as net muscle forces acting in the functional spinal unit. Specifically, ID works in an inverse sense in which the motion is *a priori* known from experimentally acquired motion capture data to derive segment body velocities and accelerations. In more advanced scenarios, normalised electromyography recordings monitoring the muscle activity for the desired movement are taken into account to assist in solving the optimisation problem in ID (Pizzolato et al., 2015; Molinaro et al., 2020; Silvestros et al., 2022).

The majority of MSK spine models today make use of the ID approach. The detailed characterisation of back muscles in Christophy et al. (2012) set the baseline in modelling of the spine upon which many current models are based (Senteler et al., 2014; Bruno et al., 2015; Dao et al., 2015; Rupp et al., 2015; Raabe and Chaudhari, 2016; Mörl et al., 2020; Overbergh et al., 2020). Bruno et al. (2015) combined, revised and completed model geometry and muscle architecture based on previous models (Vasavada et al., 1998; Christophy et al., 2012) to develop a fully articulated thoracolumbar spine model. Despite the impressive anatomical detail of the spine models presented by Christophy et al. (2012) and Bruno et al. (2015), the studies lacked physiological kinematic input. Generally, the advantage of the ID approach is that it allows analysis of advanced motion tasks such as high-impact (Cazzola et al., 2017; Silvestros et al., 2022), running (Raabe and Chaudhari, 2016), lifting (Beaucage-Gauvreau et al., 2019) or throwing activities (Molinaro et al., 2020) as well as the study of pathologic movement patterns (Overbergh et al., 2020). While current ID models of the spine are becoming increasingly complex, they face the challenge of relying on accurate and comprehensive kinematic data, which most of the current motion capture technologies cannot provide for the spine. Assessment of spinal motion from skin-mounted reflective markers is particularly difficult due to the inevitable movement artefacts prevalent on the spine during dynamic movements (Zemp et al., 2014; Mahallati et al., 2016; Papi et al., 2017). Significant efforts were made to estimate the magnitude of skin movement along the spine, however, have failed to give a clear relationship between a particular movement and the displacement of respective spine markers (Zemp et al., 2014). Moreover, the number of identifiable landmarks through palpation of the back is limited to the spinous process that on its own is not sufficient to provide reliable information on the position and orientation of the corresponding vertebra (Galbusera and Wilke, 2018; Millar et al., 2019).

As a result, for the analysis of motion tasks, the complexity of ID models is commonly drastically reduced by i) assuming interconnecting three DOF spherical joints and ii) lumping the motion of various adjacent vertebrae. The latter is typically realised by implementing linear kinematic coupling constraints (Christophy et al., 2012; Cazzola et al., 2017) further limiting the total DOF of the system, recently reviewed in Alemi et al. (2021). Furthermore, most of the current ID spine models neglect the contribution of passive joint stiffness produced by ligaments and IVDs (Christophy et al., 2012; Bruno et al., 2015; Cazzola et al., 2017; Beaucage-Gauvreau et al., 2019; Molinaro et al., 2020; Silvestros et al., 2022), thereby, highly overestimating the net muscle forces necessary to hold the spine in place.

The major aim of this study was to quantify the effect of modelling individual passive structures (i.e., ligaments and IVDs) on the remaining joint forces and torques carried by muscles in the human spine using the ID approach. For this purpose, the recently developed generic spine model (Hammer et al., 2022; Meszaros-Beller et al., 2023), implemented in the *demoa* software environment (Schmitt, 2022), was used. The thoracolumbar spine model including six DOF intervertebral joints, a detailed musculature, intersegmental ligaments and IVDs, previously used in FD simulations of a forward flexion-extension movement (Meszaros-Beller et al., 2023), was transferred into the *OpenSim* MSK modelling platform. Using the full kinematic description obtained from the FD simulations, systematic ID analysis was performed in a step-wise approach increasing the model complexity by adding individual elements and compare the ID analysis results to a standard ID “plain” model neglecting the role of spinal ligaments and IVD stiffness.

A secondary aim of this study was to compare the model performance in two different MSK modelling environments (i.e., *demoa* and *OpenSim*). For this reason, solutions for the equivalent modelling of individual structures (i.e., muscles, ligaments and IVDs) in *OpenSim* were found. Under consideration of identical geometry and soft tissue properties, the ID model kinematics and kinetics were cross-validated against FD simulation results using the *in silico* derived motion data.

The novelty of this work comprises the capability to use a sophisticated generic spine model (Meszaros-Beller et al., 2023) across two different modelling environments exploiting the strength of each environment and MB approach, e.g., the possibility to remove biological structures under the conservation of movement in the ID approach. Moreover, in a quantitative investigation, this study has shown that neglecting passive spinal structures leads to a significant overestimation of remaining joint forces and torques that muscles must balance.

## 2 Methods

### 2.1 Implementing the generic *demoa* baseline model into *OpenSim*

**FIGURE 1:**
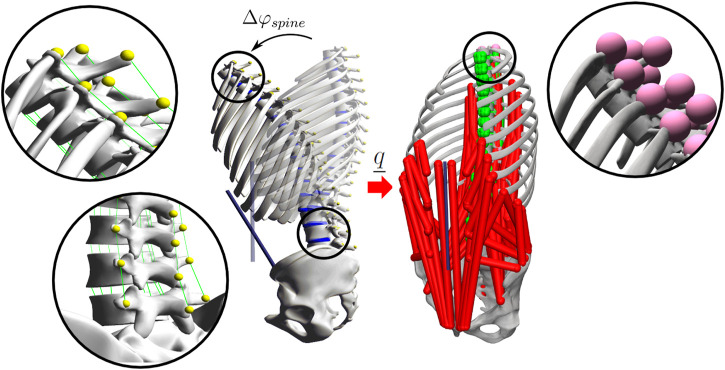
*In silico* generated motion data used for the assessment of spinal kinematics. The position of bony markers (shown in detail) was tracked during the FD simulation of a flexion-extension movement Δ*φ*_spine_ in *demoa* (left). The marker trajectories defining the precise position of each VB and resulting joint coordinates were transferred onto the identical generic baseline model implemented in *OpenSim* (right) including 102 DOF, 294 muscle fascicles (red), 208 ligaments (green), 17 intervertebral bushing elements and 51 markers (magenta). For the sake of clarity, muscles are not visualised in the *demoa* model, ligaments are shown in detail for the thoracic and the lumbar region (left).

The recently published generic baseline model (Meszaros-Beller et al., 2023) developed for the use in the *demoa* FD software environment consisting of 1) 20 rigid bodies representing the spinal anatomy, 2) 17 IVDs, 3) 192 intersegmental ligaments and 4) 294 trunk muscles was implemented into the MSK modelling platform *OpenSim* 4.3 (SimTK, Stanford, CA, United States). Figure 1 shows the generic baseline model implemented in *demoa* (left) and *OpenSim* (right). Both geometric models were generated using the in-house preprocessor *calcman*, a program for the calculation of 3D anthropometric data. Solutions for the equivalent modelling of the geometry and individual soft tissues were found and categorised into a “body set,” “joint set,” “constraint set,” “force set” and a “marker set” according to *OpenSim*’s model structure. Thereby, the joint-body structure of the model, muscle and ligament attachment points and the model’s underlying force laws maintained unchanged.

The body set and the joint set define the body properties of the model (including masses and inertia), the body frames, i.e., the relative translational and rotational offset of a body frame to the superior and inferior joint frame and the DOF prescribed to that joint. With respect to the coordinate system, the model’s body and joint position and orientation and inertial properties were transformed from the *demoa* environment to comply with the coordinate system definition in *OpenSim*. The latter is a right-handed coordinate system with the positive *x* −axis pointing anterior, the positive *y* −axis pointing cranial and the positive *z* −axis pointing right. Thus, with respect to the *demoa* coordinate system, the *OpenSim* coordinate system is rotated clockwise around the *x* − axis by 90°. In Table 1 the different axis conventions are depicted. Moreover, in both systems the positive Cardan rotations are defined to be counterclockwise around the respective axis. Consequently, a X Y Z rotation in *demoa* complies with a X (−Z) Y rotation in *OpenSim*. This must be considered in the orientation of bodies. Based on the *demoa* model, the corresponding coordinate transformation was calculated and applied to each body and joint frame to obtain the model geometry in the *OpenSim* coordinate system. Following the axis convention in Table 1, inertial effects were defined according to Eq. 1.

$${\left[\begin{array}{c} I_{xx}\;I_{xy}\;I_{xz}\\ I_{yx}\;I_{yy}\;I_{yz}\\ I_{zx}\;I_{zy}\;I_{zz} \end{array}\right]}_{\text{osim}}={\left[\begin{array}{c} I_{xx}\;I_{xz}\;-I_{xy}\\ I_{zx}\;I_{zz}\;-I_{yz}\\ -I_{yx}\;-I_{zy}\;I_{yy} \end{array}\right]}_{\text{dsim}}$$ (1)

where “osim” corresponds to the *OpenSim* and “dsim” corresponds to the *demoa* implementation as indicated by the subscript.

**TABLE 1:** Definition of coordinate systems in *demoa* and *OpenSim*.

*demoa*	*OpenSim*
X	X
Y	−Z
Z	Y
	
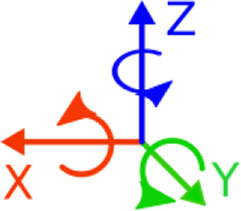	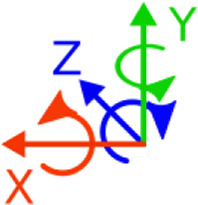

In accordance to the model described in Meszaros-Beller et al. (2023), relative motion between the pelvis and the ground as well as between the sacrum and the pelvis was inhibited by a weld joint (no DOF: fusion). Further, six DOF custom joints were defined at every vertebral level between adjacent vertebrae from the first thoracic vertebra (T1) to the sacrum (S1) allowing for three rotational and three translational DOF. The 17 individual segment masses representing the weight of each trunk slice and the linea alba representing the abdominal wall were also connected to their respective vertebral body (VB) and the last thoracic vertebra (T12), respectively, by a weld joint. Finally, gravitational effects were defined as negative in the *y* −axis according to Eq. 2.

$${\vec{g}}_{\mathrm{osim}}=\left[\begin{array}{c} 0\\ -9.81\\ 0 \end{array}\right]\frac{m}{s^{2}}$$ (2)

The “force set” included the same set of muscles, ligaments and IVDs as previously described (Meszaros-Beller et al., 2023). The coordinates of muscle and ligament attachment points were translated according to the axis convention in *OpenSim* (Table 1). In order to use the same muscle model in both the *demoa* and *OpenSim* spine model, the muscle’s activation and contraction dynamics according to Rockenfeller and Günther (2018) and Häufle et al. (2014) have been implemented as a new functionality into *OpenSim* and used as a plugin. Note, despite that muscles were implemented, the focus of the present study lied in the ID analysis of the *OpenSim* model, i.e., the computation of generalised net joint forces and torques as a result of all biological model structures implemented neglecting the contribution of muscles. The process of distributing the ID-derived generalised net joint loads onto individual muscle fascicles, in the framework of the so-called static optimisation (SO), is subject to further investigation and will be the focus of future work.

Intersegmental ligaments were implemented as straight line elements applying a length-dependent tensile force using *OpenSim*’s “SimmSpline” function. Thirteen points were selected on the individual force-length curve of each ligament (Meszaros-Beller et al., 2023, Section 2.1.4) with the length-values scaled by the ligament rest-length *l* ^LIG,0^ and the force-values scaled by the parameter *F*_*B*_, the force component of the characteristic point B(*ɛ*_*B*_, *F*_*B*_) (state before failure at strain *ɛ*_*B*_) of the parametrised ligament model used in *demoa* (Günther et al., 2007). Note, as the non-linear ligament force function in the *demoa* model allows increasing forces beyond B (*ɛ*_*B*_, *F*_*B*_), the ligament force-strain data in the *OpenSim* model were, therefore, linearly extended to include data points at 1.5 ⋅*ɛ*_*B*_ and 2.0 ⋅*ɛ*_*B*_ accounting for potential overloading of ligaments. Given the same ligament rest-lengths were used as in the previously presented *demoa* model (Meszaros-Beller et al., 2023, Section 2.1.4), ligament pre-strain was also accounted for in the *OpenSim* model.

In accordance with the *demoa* model, IVDs were implemented as expression-based bushing elements and set equal to the respective joint frame. The same stiffness parameters were used as previously defined (Meszaros-Beller et al., 2023, Table 1) except for the lumbar stiffness in lateral bending that was reset to the original literature value of 93 Nm/rad. The rationale for this was that the generic baseline model used in this study is symmetric in the sagittal plane and does not require lateral reinforcement. Similarly, the effect of intrinsic IVD pressure was also considered by adding an uni-directional prestrain–the constant offset force *F* ^IVD,0^ pointing along the local *y*−axis of the joint reference frame–that was estimated from the weight of cumulated VB and segment masses located proximally to each joint (Meszaros-Beller et al., 2023). Thus, the total IVD force *F* ^IVD^ along the local *y*−axis is a superposition of forces according to Eq. 3.

$$F^{\text{IVD}}=F^{\text{IVD,stiff}}+F^{\text{IVD,0}}$$ (3)

with *F* ^IVD, stiff^ representing the state-dependent bushing element force. No kinematic constraints were applied between the bodies.

Note, particular attention was paid to the identical definition of geometric and soft tissue properties with respect to decimal digits. This is important as slight inconsistencies in decimal digits between the *demoa* and *OpenSim* model might lead to deviations in kinematics and the force response by individual biological structures.

### 2.2 *In silico* motion data for model kinematics

*In silico* motion data (marker trajectories) for a flexion-extension movement were obtained from FD simulations in *demoa* v2.2 (http://get-demoa.com), as previously described (Meszaros-Beller et al., 2023, Section 2.3). The generic baseline model (medium co-contraction level: $u_{\text{open}}^{\text{abd}}=0.02$ , $u_{\text{open}}^{\text{back}}=0.04$ ) equipped with 51 virtual markers, i.e., three markers per VB corresponding to existing ligament and muscle attachment points on the spinous process and on the left and right transverse process of each vertebra (Figure 1) was used to rerun the muscle-driven FD simulations in *demoa*. The marker positions were tracked over the simulation time *t*_SIM_ in global coordinates resulting in a complete, gap-free motion data set. The generated *in silico* motion data started in an equilibrated state under gravitational load.

In contrast to the previously presented model (Meszaros-Beller et al., 2023), in this study, the pelvic tilt as well as the ligament and IVD damping was set to zero. Further, the muscles’ straight line elements between insertion and origin points were redirected by via-points instead of via-ellipses.

Identical markers were defined in the generic *OpenSim* spine model that was used together with the resulting *in silico* motion data to perform an inverse kinematics (IK) analysis in *OpenSim*. Given no marker errors were present, marker weights for all markers were set equal to 1. In Figure 1 the transfer of *in silico* motion data from the *demoa* model to the *OpenSim* model is visualised. The resulting ID joint coordinates were compared to individual joint angles obtained from the FD simulation in *demoa* in order to confirm identical model kinematics.

### 2.3 Inverse-dynamics analysis

Systemic ID analysis was performed using OpenSim 4.3 GUI and the Häufle muscle model (Häufle et al. (2014)) plugin through the MATLAB API. Given ID analysis provides the generalised net joint forces and torques as a result of all biological model structures implemented, a step-wise approach according to Table 2 was used in order to obtain the individual contribution of each structure. For this purpose, the generic *OpenSim* model was discretised into five “feature” models with increasing complexity starting with i) a “plain” model including VB and segment masses; ii) a “LIG” model including VB and segment masses and ligaments; iii) an “intrinsic IVD” model including VB and segment masses and the IVD offset force *F* ^IVD,0^ in local axial direction; iv) a “full IVD” model including VB and segment masses, and the full IVD force (see Eq. 3); and v) an “all element” model including VB and segment masses, ligaments and the full IVD.

**TABLE 2:** ID analysis scenarios.

Feature	Plain	LIG*	Intrinsic IVD	Full IVD*	All elements
VB mass	*✓*	*✓*	*✓*	*✓*	*✓*
Segment mass	*✓*	*✓*	*✓*	*✓*	*✓*
Ligaments		*✓*			*✓*
IVD offset force			*✓*	*✓*	*✓*
IVD stiffness				*✓*	*✓*

Note, the “plain” model represents the standard ID model available in literature. In the “intrinsic IVD” model, the IVD stiffness was set equal to zero in all directions except for the constant unidirectional offset force $F^{\text{IVD,0}}$ in axial direction. If considered, the IVD stiffness was applied in all six DOF. *The “LIG” and “full IVD” model marked with an asterisk were used to cross-validate the contribution of ligaments and IVDs.

It is worth mentioning that the “plain” model reflects the structural complexity of passive tissue elements in the majority of current ID spine models available in literature (Christophy et al., 2012; Bruno et al., 2015; Cazzola et al., 2017; Beaucage-Gauvreau et al., 2019; Molinaro et al., 2020; Silvestros et al., 2022). Each of the five “feature” models i) - v) underwent ID analysis using the same kinematic input for a flexion-extension movement as described in Section 2.2.

The contribution of ligaments and IVDs to the generalised joint loads was evaluated by computing the difference between remaining axial joint forces $F_{y,i}^{*}$ and torques $T_{z,i}^{*}$ in the sagittal plane and the generalised axial joint loads of the “plain” model, $F_{y,i}^{\text{plain}}$ and $T_{z,i}^{\text{plain}}$ , according to Eqs 4, 5.

$$\Delta F_{y,i}^{*}=F_{y,i}^{*}-F_{y,i}^{\text{ plain}}$$ (4)

$$\Delta T_{z,i}^{*}=T_{z,i}^{*}-T_{z,i}^{\text{ plain}}$$ (5)

where the subscript *i* corresponds to the vertebral joint from L1/2 to L5/S1 and the asterisk corresponds to the “LIG” model and the “full IVD” model, respectively, marked in Table 2. To cross-validate the individual structural contribution, the obtained results were then compared to the internal loads predicted in the FD simulation in *demoa*.

## 3 Results

IK and ID analysis was performed using the generic *OpenSim* spine model with and without the contribution of individual structures according to Table 2 using *in silico* motion data for a flexion-extension movement. For the interpretation of ID analysis results, the reader is referred to Supplementary Figure 1.

### 3.1 Model kinematics

**FIGURE 2:**
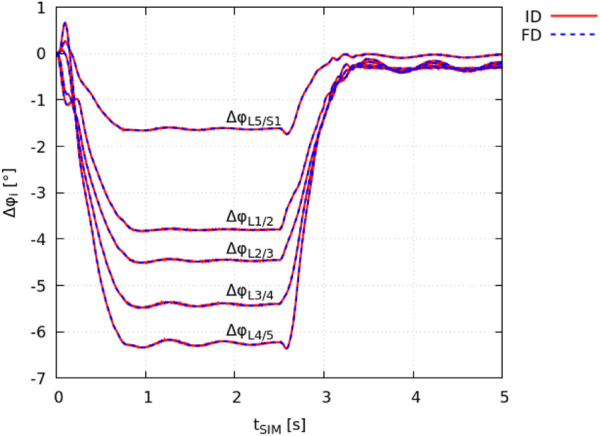
Validation of identical model kinematics. The change in intervertebral joint angles Δ*φ*_*i*_, obtained from *demoa* with respect to the model’s equilibrated state (FD: blue dashed line) and *OpenSim* (IK: red solid line) is visualised for individual lumbar joints *i*.

The ID joint kinematics complied with the joint angles obtained in the FD simulation as a result of the prescribed motion data (Section 2.2). The total squared error and maximum marker error was 3.1 ⋅ 10^–9^ m^2^ and 4.5 ⋅ 10^–5^ m (root mean square error of 7.8 ⋅ 10^–6^ m), respectively. The absolute individual joint angles at *t*_SIM_ = 0 *s* had a maximum deviation of ± 0.008° from the FD simulation (equilibrated state). In Figure 2, the change in individual lumbar joint angles Δ*φ*_*i*_ during the flexion-extension movement is shown for both the ID and FD approach.

It is noted that the peak spinal flexion is reached at *t*_SIM_ ≈ 1 *s*. The flexed position is held for 1.5 *s* followed by the extension movement that is completed at *t*_SIM_ ≈ 3.5 *s*.

### 3.2 ID analysis by step-wise increasing model complexity

**FIGURE 3:**
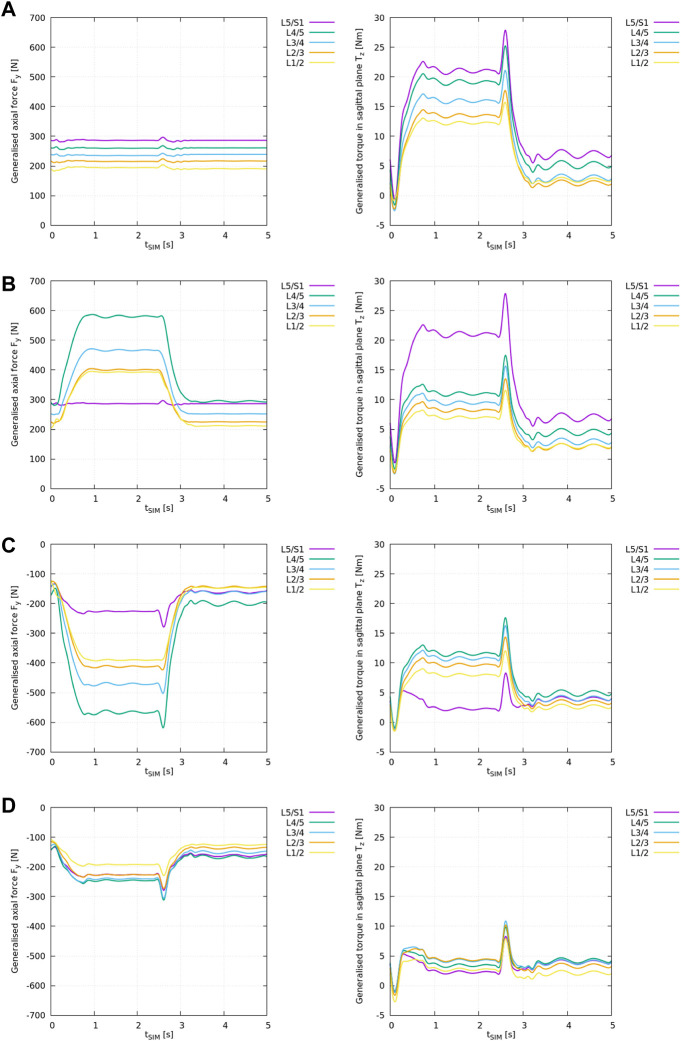
ID analysis of different “feature” models according to Table 2 with the generalised axial force *F*_*y*,*i*_ (left) and torque in the sagittal plane *T*_*z*,*i*_ (right) for all lumbar levels *i* from L1/2 to L5/S1. **(A)** ID analysis of the “plain” model, **(B)** ID analysis of the “LIG” model, **(C)** ID analysis of the “full IVD” model, **(D)** ID analysis of the “all elements” model.

The results from the ID analysis (Table 2) are shown in Figure 3 and elucidated systematically in the following.

Note, in the interpretation of ID results one needs to consider that the ID tool in *OpenSim* outputs the required net muscle forces and torques to compensate for the generalised joint loads, or, in case of modelled soft tissue elements, to compensate for the remaining joint loads. Thus, a negative axial force *F*_*y*,*i*_ for joint *i* in Figure 3 means that the remaining joint forces are decompressive, i.e., existing muscles act in compression (physiologically possible). A positive axial force, on the other hand, means that the remaining joint forces are compressive, i.e., existing muscles would need to apply positive (pushing) forces which is physiologically impossible (see Supplementary Section 1).

Similarly, a positive torque *T*_*z*,*i*_ in the sagittal plane implies an anterior joint loading that requires the muscles to produce a positive (posterior) torque according to *OpenSim*’s axis convention (Table 1). The reader is referred to Supplementary Figure 1 for more details.

#### 3.2.1 Neglecting all passive structures

The results from the “plain” model ID analysis are shown in Figure 3A. Compressive joint forces and anterior joint torques increased caudally.

With flexion, the generalised axial forces *F*_*y*,*i*_ (Figure 3A: left) stayed nearly constant while the generalised torque component (Figure 3A: right) increased at all lumbar levels.

Note, the generalised axial forces complied with the weight of the respective cumulated body and segment masses as these are the only forces acting onto the joints in the “plain” scenario (see Supplementary Figure 1).

#### 3.2.2 Considering passive net ligament contribution

The results from the “LIG” model ID analysis are shown in Figure 3B.

With respect to the “plain” model, the inclusion of ligaments changed the axial loading pattern in *F*_*y*,*i*_ from a nearly constant compressive loading (Figure 3A: left) to an increasing compressive loading of the remaining joint forces at all levels *i* with spinal flexion (Figure 3B: left) except at level L5/S1 where no ligaments were included. Due to the superposition of gravitational and ligament forces acting onto the joint (see Supplementary Figure 1), compressive joint loading increased on average by +102% between L1/2 and L4/5.

Moreover, with respect to the “plain” model, the inclusion of ligaments also reduced the remaining anterior joint torques *T*_*z*,*i*_ (Figure 3B: right) on average by −41% between L1/2 and L4/5 except at level L5/S1 where no ligaments were included.

#### 3.2.3 Considering passive IVD contribution

The results from the “full IVD” model ID analysis are shown in Figure 3C.

With respect to the “plain” model, the inclusion of linear IVD stiffness properties and the axial offset force changed the axial loading pattern in *F*_*y*,*i*_ from a nearly constant compressive loading of the remaining joint forces (Figure 3A: left) to an increasing decompressive joint loading of the remaining joint forces at all levels *i* with spinal flexion (Figure 3C: left). This reflects the counteracting role of this cartilaginous tissue, compensating for all compressive forces in the spine as depicted in Supplementary Figure 1. Consequently, remaining compressive joint loading decreased on average by −302% between L1/2 and L4/5 and by −179% at L5/S1. Thereby, this is the first model variant in which the interpretation of ID results as net muscle forces is meaningful.

Moreover, with respect to the “plain” model, the inclusion of IVD stiffness reduced the remaining anterior joint torques *T*_*z*,*i*_ on average by −34% between L1/2 and L4/5 and by −89% at L5/S1.

Note, the results from the “intrinsic IVD” model ID analysis are displayed and elucidated in Supplementary Section 2.

#### 3.2.4 Considering the contribution of all passive elements

The results from the “all elements” model ID analysis are shown in Figure 3D.

With respect to the “plain” model, the inclusion of IVDs and ligaments together changed the axial loading pattern in *F*_*y*,*i*_ from a nearly constant compressive joint loading (Figure 3A: left) to an increasing decompressive joint loading of the remaining joint forces at all levels *i* with spinal flexion (Figure 3D: left). Compressive joint loading decreased on average by −200% between L1/2 and L4/5 and by −179% at L5/S1.

Moreover, with respect to the “plain” model, the inclusion of IVDs and ligaments together reduced the remaining anterior joint torques *T*_*z*,*i*_ on average by −75% between L1/2 and L4/5 and by −89% at L5/S1. Compared to the “full IVD” model, the remaining forces and torques decreased markedly, and, consequently, also the assumed net muscle forces.

### 3.3 Cross-validation of individual structural contribution

The individual structural contribution of ligaments and IVDs to the generalised net axial force (*F*_*y*,*i*_) and torque in the sagittal plane (*T*_*z*,*i*_) was computed according to Eqs 4, 5.

**FIGURE 4:**
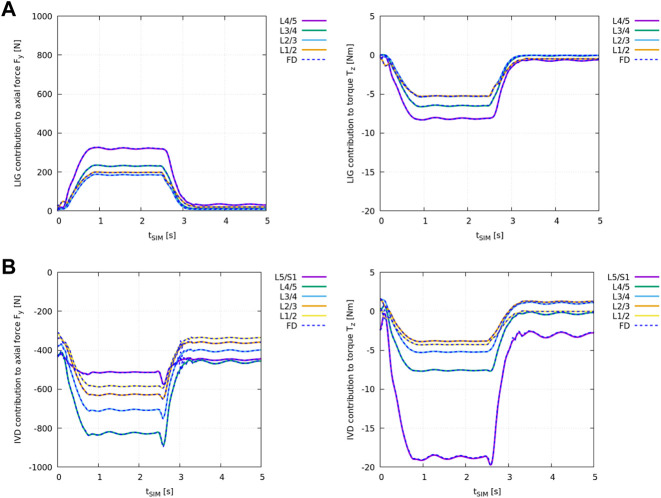
Individual structural contribution of ligaments in **(A)** and IVDs in **(B)** to the axial force *F*_*y*,*i*_ (left) and the torque in the sagittal plane *T*_*z*,*i*_ (right) for all lumbar joints. Ligaments produce a tensile (positive) force and an anterior (negative) torque while IVDs are under compression and apply a (negative) force and an anterior (negative) torque with forward flexion. Given relative values of remaining joint loads were compared, the loading experienced by the individual structure is displayed in *OpenSim*’s axis convention. The blue dashed lines show the results obtained from the FD simulation in *demoa*. Note, for the visualisation of *T*_*z*,*i*_, the FD results were mirrored on the time-axis due to the opposite definition of positive rotation (Table 1). Note, no ligaments were implemented at level L5/S1 (see Meszaros-Beller et al., 2023, Section 2.1.4).

Figure 4A shows the difference in ID analysis results between the “plain” model and the “LIG” model (Table 2) compared against the individual net ligament contribution obtained from the FD simulation in *demoa* (blue dashed line).

Figure 4B shows the difference in ID analysis results between the “plain” model and the “full IVD” model (Table 2) compared against the individual IVD contribution obtained from the FD simulation in *demoa* (blue dashed line).

Both, ligament and IVD contribution complied between the ID and FD approach at all states.

## 4 Discussion

Current ID models of the spine (Christophy et al., 2012; Bruno et al., 2015; Cazzola et al., 2017; Beaucage-Gauvreau et al., 2019; Molinaro et al., 2020; Silvestros et al., 2022) commonly face two major limitations that is i) the lack of accurate kinematic data and ii) the absence of passive elements, i.e., ligaments and IVDs. While the latter can be solved by modelling the individual structures or by the incorporation of a “lumped” joint stiffness (Wang et al., 2020), the accurate and reliable measurement of spinal motion is made challenging due to skin movement artefacts, marker misplacement (Papi et al., 2017) and the lack in identifiable bony landmarks from the back that is limited to the spinous process (Galbusera and Wilke, 2018). As a result, commonly gross spinal motion is recorded and applied on models with reduced model complexity, e.g., using simplified three DOF spherical joints and linear kinematic coupling constraints for the thoracic and lumbar spinal region (Alemi et al., 2021), respectively. These simplifications, however, are in conflict with the increasing complexity of spine models and introduce intrinsic errors in the computation of kinematics that may propagate to even larger errors in the computation of joint loads and muscle forces (Byrne et al., 2020; Alemi et al., 2021). Currently there is no solution to this problem.

In the present study, *in silico* motion data obtained from a FD simulation allowed to perform ID analysis of a detailed fully articulated thoracolumbar spine model with six DOF joints avoiding error-prone experimental data. For this purpose, the recently developed generic baseline model (Meszaros-Beller et al., 2023) was transferred with respect to its geometric and soft tissue properties into an established MSK modelling platform, *OpenSim* 4.3. Note that the FD simulation kinematics might differ from human spine flexion. However, due to the aforementioned limitations, experimental datasets are not suited for this comparative approach yet.

The minimal cross-platform losses in accuracy due to the transformation of rotations from degrees to radians of ± 0.008° were considered negligible. The advantage of using *in silico* motion data over experimental marker-based motion data typically acquired in a motion capture laboratory is that one has access to precise intervertebral kinematics that is not biased by common limitations of marker-based motion capture techniques or any post processing, i.e., filtering and gap-filling. Three markers per vertebra were successfully tracked over the course of the flexion-extension motion in the FD simulation in *demoa* and used to run IK analysis on the same model implemented in *OpenSim*. Given the motion data were obtained from the same model, no marker error was present. In Figure 2, the identical model kinematics were verified via comparison of individual lumbar joint angles between the FD and ID approach.

In addition to the difficulty in obtaining accurate kinematic data described above, the majority of ID spine models neglect passive elements such as ligaments and IVDs that contribute with a posterior torque component to the system dynamics counteracting the predominant anterior loading of the spine (Supplementary Figure 1). As a consequence, neglecting passive elements results in an overestimation of remaining joint loads and, subsequently, predicted net contribution of muscle forces to the joint force and torque. At the same time, commonly employed optimisation processes are known to underestimate muscle co-contraction (Cazzola et al., 2017; Beaucage-Gauvreau et al., 2019).

For the first time, this study assessed the effect of individual passive structures (i.e., ligaments and IVDs) on the ID analysis results using FD-derived *in silico* kinematics in a step-wise approach increasing the model complexity (Table 2). As shown in Figures 3A–D, the incorporation of ligaments and/or IVDs changed the magnitude and the pattern of the ID results. Thus, it can be concluded that passive elements likely affect predicted muscle recruitment patterns. Typically, this is obtained through static optimisation (SO), an optimisation process following the ID analysis in which the ID-derived generalised net joint forces and torques, or remaining loads after consideration of passive soft tissue elements are distributed to individual muscle fascicles. Even though, SO analysis lied beyond the scope of this study and individual muscle contribution were not examined here, it will be considered in future work. However, the muscle forces used to produce the spine kinematics are presented in Meszaros-Beller et al. (2023) and raw data is available from Hammer et al. (2022).

It is worth noting that the simplest model analysed in this study, i.e., the “plain” model neglecting all passive structures was representative for the standard ID spine models currently available in literature. If only the VB and segment masses were incorporated in the spine model, the corresponding ID results in Figure 3A imply the patently wrong statement that with forward flexion muscles would have to push and pull at the same time in order to withstand gravitational load and the anterior torque generated through the upper body. Without additional decompressive elements, the joints would need to be simplified to three DOF spherical joints inhibiting translational joint movement to avoid this problem, see Supplementary Section 1.

Regarding the IVD and ligament implementation in *OpenSim* and *demoa*, ID-derived structural contribution was cross-validated at every lumbar level against FD simulation results to ensure their identical implementation. Both ligaments (Figure 4A) and IVDs (Figure 4B) contributed equally to the net joint axial force *F*_*y*_ and the net joint torque in the sagittal plane *T*_*z*_ in the FD and ID approach over the course of the flexion-extension movement.

With respect to the ID analysis results, ligaments and IVDs both significantly affected the estimated remaining joint loading (Figures 3B–D): Between L1/2 and L4/5 the modelling of passive structures reduced both net contribution of muscle forces, compressive loading and anterior torque, on average by −200% and −75%, respectively. The relatively high joint decompression through passive elements can be partly attributed to the IVD offset force $F^{\text{IVD,0}}$ that was responsible for a −101% reduction in compressive loading entirely compensating for the gravitational load as intended. These results demonstrate that passive structures contribute to a redistribution of spinal loading among the different structures and should not be omitted in MSK spine models.

As anticipated, the implementation of ligaments and IVDs, respectively, had an opposite effect on the axial loading which can be explained through their line of action: while IVDs are ‘pushing’ vertebrae away from each other under compression, ligaments act in the opposite direction and restrain their ‘sliding apart’ through tensile forces (see Supplementary Figure 1). Thereby ligaments act in line with the muscles. This is in accordance with the results presented.

Furthermore, the presented net ligament contribution to the axial force reveals the strong compressive force onto the IVDs which would be underestimated in a spine model without ligaments included. Thus, considering ligaments does not only affect calculated remaining joint loads and corresponding net muscle forces in the ID analysis but already the predicted bone-on-bone force (Winter 2009) in FD simulations. In our case, this force acting onto the cartilage between bony segments comprises only the IVD loads. Its estimation has the highest clinical relevance of all modelled soft tissue elements in this study, e.g., for the design and development of biomaterials for IVD replacement.

The validity of the “all elements” spine model presented in this study was previously discussed (Meszaros-Beller et al., 2023, Section 4.1). Even though the structural complexity of the “all elements” spine model is one rarely seen among ID spine models, limitations include the absence of the intra-abdominal pressure, the limited modelling of the ribcage, and the rudimentary modelling of the facet joints (Meszaros-Beller et al., 2023, Section 4.1). In the future, the presented model can be used for various comparative studies, e.g., exploring different neural control solutions and muscle recruitment patterns employed in the FD and ID approach. Further, by having access to precise kinematics the inaccuracies introduced through kinematic coupling constraints (Alemi et al., 2021) could be evaluated. The work presented in this study cross-validated the kinematics and kinetics of the developed generic spine model and its implementation in *OpenSim* providing the necessary baseline for further investigation.

## 5 Conclusion

Summarising, the presented approach has demonstrated the capability of cross-platform analysis of an identical detailed generic spine model by using the FD and ID approach avoiding common limitations in motion capture. Moreover, the used step-wise approach allowed us to quantitatively investigate the effect of passive structures on the ID results. IVDs and ligaments together have shown to significantly reduce remaining compressive joint loading implicating reduced muscle forces needed to balance the net joint forces and torques. This work provides the necessary baseline towards exploring different neural control solutions employed in the FD and ID approach.

## Data Availability

The raw data supporting the conclusion of this article will be made available by the authors, without undue reservation.
