# Supplementary material for: Effect of neglecting passive spinal structures: a quantitative investigation using the forward-dynamics and inverse-dynamics musculoskeletal approach
Source: Front Physiol. 2023 May 31;14:1135531. doi: 10.3389/fphys.2023.1135531 (PMC10264677; doi:10.3389/fphys.2023.1135531)
Supplement: Supplementary file 1 [file DataSheet1.pdf]

Supplementary Material

Effect of neglecting passive spinal structures: a quantitative investigation using the forward-dynamics and inverse-dynamics musculoskeletal approach

Frontiers in Physiology

Laura Meszaros-Beller[1,2,3*], Maria Hammer[3,4], Syn Schmitt[1,3,4*] and Peter Pivonka[1,2*]

[1]School of Mechanical, Medical and Process Engineering, Queensland University of Technology, Brisbane, Australia.
[2]Centre for Biomedical Technologies, Queensland University of Technology, Brisbane, Australia.
[3]Institute for Modelling and Simulation of Biomechanical Systems, University of Stuttgart, Germany.
[4]Stuttgart Center for Simulation Science (SC SimTech), University of Stuttgart, Germany.

*Corresponding author(s). E-mail(s): l2.meszaros@qut.edu.au; schmitt@simtech.uni-stuttgart.de; peter.pivonka@qut.edu.au;

Contents

1 Forces and torques acting on the spinal motion segment **2**

2 ID analysis using the "intrinsic IVD" model **2**

1 Forces and torques acting on the spinal motion segment

The musculoskeletal (MSK) system of the spine comprises bones (e.g., vertebrae) connected by joints, muscles, and passive structures such as ligaments and the intervertebral disc (IVD) that significantly contribute to the total net joint forces and torques. Thus, with respect to the general equation of motion in multibody dynamics [1], the force $f_i(q,\dot{q})$ and torque $\tau_i(q,\dot{q})$ acting on a joint i can be calculated according to Eqs 1, 2.

$$f_i(q,\dot{q}) = F_i^E(q,\dot{q}) + \sum_m F_m^{\text{MTU}}(q,\dot{q}) + \sum_n F_n^{\text{LIG}}(q) + F_i^{\text{IVD}}(q) \quad (1)$$

$$\tau_i(q,\dot{q}) = T_i^E(q,\dot{q}) + \sum_m R_{m,i}^{\text{MTU}}(q) F_m^{\text{MTU}}(q,\dot{q}) + \sum_n R_{n,i}^{\text{LIG}}(q) F_n^{\text{LIG}}(q) + T_i^{\text{IVD}}(q) \quad (2)$$

where q, $\dot{q}$ are the vectors of generalised joint coordinates and velocities, respectively; $F_m^{\text{MTU}}(q,\dot{q})$ and $F_n^{\text{LIG}}(q)$ are the vectors of musculotendon and ligament forces for all muscles m and all ligaments n spanning the intervertebral joint i; $R_{m,i}^{\text{MTU}}(q)$ $R_{n,i}^{\text{LIG}}(q)$ are the corresponding muscle and ligament moment arms, and $R_{m,i}^{\text{MTU}}(q)F_m^{\text{MTU}}(q,\dot{q})$ and $R_{n,i}^{\text{LIG}}(q)F_n^{\text{LIG}}(q)$ are the resulting torque contributions to the joint load; $F_i^{\text{IVD}}(q)$ and $T_i^{\text{IVD}}(q)$ are the IVD force and torque vectors of IVD torques; and $F_i^E(q,\dot{q})$ and $T_i^E(q,\dot{q})$ are the vectors of external forces and torques acting onto the joint, which only contain gravitational loads in this study. Note, in general, IVD and ligament forces do also depend on $\dot{q}$. In this study, however, mechanical damping of these two passive tissue elements was neglected.

Fig. 1 shows all forces and torques acting on a spinal motion segment under the assumption of a six degree of freedom (DOF) joint. Note, in inverse-dynamics (ID) analysis the contribution of muscles (Fig. 1: red) is neglected.

Considering the standard "plain" model stripped by all IVD and ligament forces, the force equilibrium would require muscles to produce a positive (pushing) force according to $-F^{\text{MTU}} \approx F_i^{\text{S}} + F_i^{\text{VB}}$. Here, $F^{\text{MTU}} = \sum_m F_m^{\text{MTU}}$ and F_i^{S} and F_i^{VB} represent the gravitational forces of the summed segment and vertebral body (VB) weights, respectively, which are located superior to joint i. For the torque balance, the muscles would need to produce a positive (posterior) net torque, i.e., by applying a negative (pulling) force, to counteract the negative (anterior) torque created by the VB T_i^{VB} and segment weight T_i^{S} according to $-T^{\text{MTU}} \approx T_i^{\text{S}} + T_i^{\text{VB}}$ with $T^{\text{MTU}} = \sum_m R_{m,i}^{\text{MTU}} F_m^{\text{MTU}}$. Given muscles can only act in compression through muscle contraction, this inconsistency is typically solved by constraining the translational DOFs in intervertebral joints and neglecting the force balance.

On the other hand, considering the "all elements" model, the modelled passive elements in sum are decompressive, and can, thus, compensate for the pulling forces of muscles.

2 ID analysis using the "intrinsic IVD" model

IVD contribution to the generalised axial joint force $F_{y,i}$ and torque in the sagittal plane $T_{z,i}$ was evaluated in two steps. First, the level-specific offset force $F^{\text{IVD},0}$ in axial direction was applied using the "intrinsic IVD" model. Second, the six DOF stiffness matrix was included in the "full IVD" model, see Table 2 and Figure 3 in the main text for reference. The results from the "intrinsic IVD" model ID analysis are shown in Fig. 2. With respect to the "plain" model, the inclusion of the IVD offset forces uniformly reduced the constant compressive loading of the "plain" model on average by -101% between L1/2 and L5/S1 such that $0\ N > F_{y,i} > -7\ N$. This is expected, given the IVD offset forces were estimated from the weight of cumulated VB and segment masses located proximally to each joint.

Applied as a constant unidirectional force along the local axial direction of each IVD, $F^{\text{IVD},0}$ did not have an effect on the remaining anterior joint torques $T_{z,i}$.

References

[1] Pandy MG. Computer modeling and simulation of human movement. Kinesiology. 2001;3(1):245–273. https://doi.org/10.1146/annurev.bioeng.3.1.245.

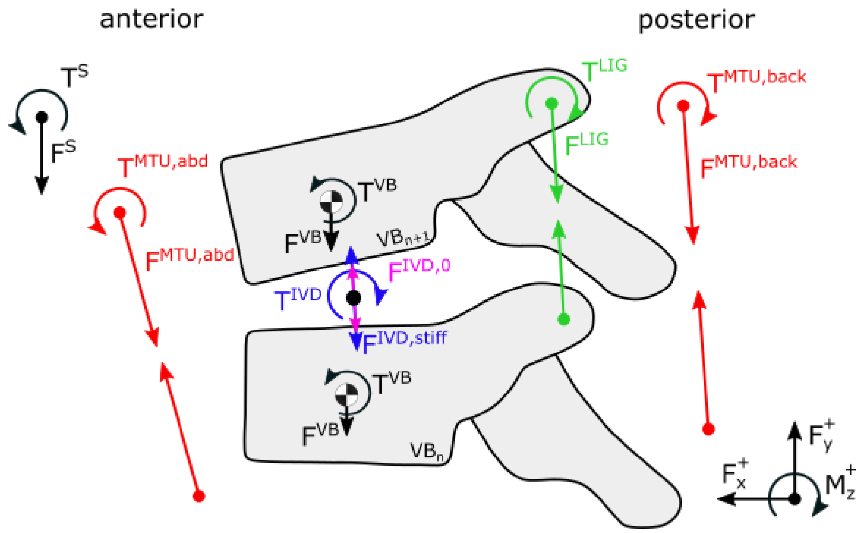

Fig. 1: Forces and torques acting on the spinal motion segment under the assumption of a six DOF joint. The weight of the cumulated VB and segment masses pointing downwards create a negative (compressive) joint force ($F^{\mathrm{VB}} + F^{\mathrm{S}}$) and a negative (anterior) joint torque ($T^{\mathrm{VB}} + T^{\mathrm{S}}$) with respect to the axis convention indicated. The IVD bushing element counteracts the anterior torque of the motion segment by introducing a positive (posterior) torque T^{IVD}. In addition to T^{IVD}, the total IVD force F^{IVD} (see Eq. 3 in the main text) is applied such that $-F^{\mathrm{IVD},0} \approx F^{\mathrm{S}} + F^{\mathrm{VB}}$. The IVD forces ($F^{\mathrm{IVD,stiff}}$: blue, $F^{\mathrm{IVD},0}$: purple) are applied at the intervertebral joint position with opposite signs onto adjacent VBs. The ligaments (green) and muscles (red) together further counteract the anterior torque such that $-F^{\mathrm{IVD,stiff}} \approx F^{\mathrm{LIG}} + F^{\mathrm{MTU,back}} + F^{\mathrm{MTU,abd}}$ by pulling on the superior vertebra downwards. Note, given a six DOF joint accounting for translational movements is used, no joint reaction forces are present. The load created by the upper body with respect to the joint is carried by surrounding tissue. Note that depicted force arrows are not to scale.

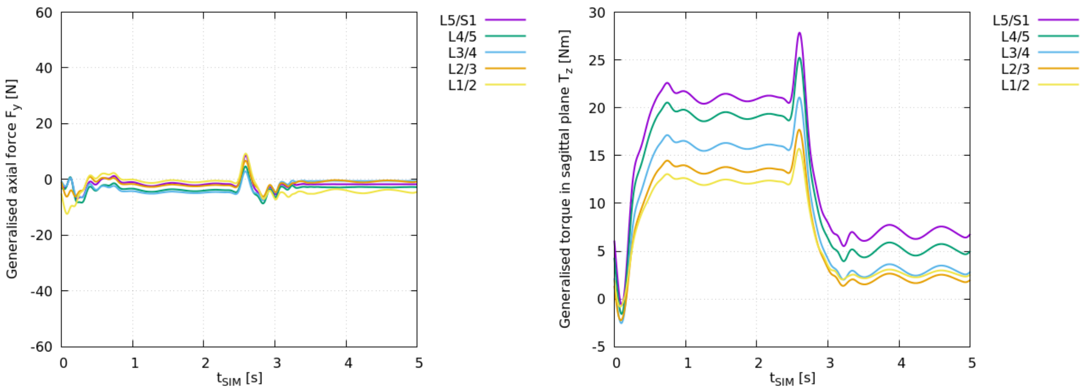

Fig. 2: ID analysis of the "intrinsic IVD" model according to Table 2 in the main text with the generalised axial force $F_{y,i}$ (left) and torque in the sagittal plane $T_{z,i}$ (right) for all lumbar levels i.
